# Domestication‐associated reduction of methyl salicylate in tomato root and its significance for resistance to root‐knot nematode

**DOI:** 10.1111/nph.71478

**Published:** 2026-07-29

**Authors:** Weijiao Wang, Manoj Sapkota, Sobhan Bahrami Zadegan, Tarek Hewezi, Esther van der Knaap, Feng Chen

**Affiliations:** ^1^ Department of Plant Sciences University of Tennessee Knoxville TN 37996 USA; ^2^ Department of Horticulture University of Kentucky Lexington KY 40546 USA; ^3^ Department of Horticulture and Institute of Plant Breeding, Genetics & Genomics University of Georgia Athens GA 30602 USA

**Keywords:** domestication, *Meloidogyne incognita*, methyl salicylate, root volatiles, SABATH methyltransferase, *Solanum lycopersicum*

## Abstract

- Methyl salicylate (MeSA) plays diverse roles in the aerial parts of plants. By contrast, its biosynthesis and function in roots remain poorly understood.
- Here, we investigated root MeSA biosynthesis and function in tomato. Genome‐wide association studies (GWAS) were performed using root MeSA levels as the phenotype in a diversity panel of 167 accessions to identify associated loci. Candidate genes were biochemically characterized, and the role of MeSA in defense against root‐knot nematode (RKN, *Meloidogyne incognita*) was evaluated using transgenic plants.
- MeSA was identified as a major root volatile in tomato and showed a domestication‐associated reduction. GWAS revealed multiple loci associated with natural variation in root MeSA, including a major locus on Chromosome 9 encoding the salicylic acid methyltransferase (SlSAMT). *SlSAMT*‐overexpressing plants showed reduced resistance to RKNs, whereas *SlSAMT*‐knockdown plants exhibited enhanced resistance.
- Our results suggest complex roles of MeSA and the salicylic acid (SA) signaling pathway in belowground plant defense. The SA signaling pathway likely plays critical roles in protecting roots against diverse natural enemies, including RKNs. Nevertheless, RKNs appear to have co‐opted MeSA as a host‐location signal, and the domestication‐associated reduction of root MeSA in tomato has likely contributed to enhanced resistance against RKNs.

## Graphical abstract

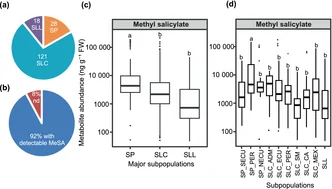

## Introduction

Salicylic acid (SA) is a central regulator of plant immunity, orchestrating defense responses against a wide range of pathogens and herbivores (Ding & Ding, 2020; Peng *et al*., 2021). Recent advances have further refined our understanding of SA biosynthesis and regulatory networks (Gong *et al*., 2023; Liu *et al*., 2025; Wang *et al*., 2025; Zhu *et al*., 2025), highlighting the significance of SA and its derivatives in plant defense. Methyl salicylate (MeSA), a methylated derivative of SA, is a typical herbivore‐induced plant volatile (HIPV) (Li *et al*., 2024) and functions as an essential mobile signal mediating long‐distance communication in systemic acquired resistance (SAR): Upon transport to distal tissues, MeSA can be converted back to SA to activate defense responses, thereby enabling the transmission of defense signals between local and distal tissues (Park *et al*., 2007; Vlot *et al*., 2008, 2009). Recent studies have provided a breakthrough in understanding the molecular basis of airborne MeSA perception, revealing a signaling module that mediates volatile‐triggered defense responses (Gong *et al*., 2023). More broadly, MeSA, as one of the most prominent defense‐related volatile organic compounds (VOCs), mediates diverse interactions between plants and their environment (Gondor *et al*., 2022), including plant–plant communication, herbivore interactions, indirect defense responses, and reproductive processes (Ulland *et al*., 2008; Singewar *et al*., 2021; Riahi *et al*., 2022; Ben‐Zvi *et al*., 2026). Despite these advances, current understanding of MeSA functions is largely focused on aerial tissues, leaving its roles in belowground environments poorly understood.

At the biochemical level, MeSA is produced through methylation of SA catalyzed by SA methyltransferases (SAMTs) belonging to the SABATH family (Ross *et al*., 1999; D'Auria *et al*., 2003). Members of the SABATH family have undergone extensive functional diversification during plant evolution, giving rise to enzymes with distinct substrate specificities toward a wide range of phytohormones and secondary metabolites (Wang *et al*., 2024). Notably, SAMTs represent one of the most frequently and independently evolved functional groups within the SABATH family, with multiple lineage‐specific origins across bryophytes, gymnosperms, and angiosperms (Dubs *et al*., 2022; Wang *et al*., 2024), highlighting the functional importance of SAMT‐mediated MeSA as a key metabolite in plant adaptation and environmental interactions. Consistently, previous studies in aerial tissues have shown that modulation of SAMT activity can influence SA‐mediated defense responses in plant immunity (Koo *et al*., 2007; Zou *et al*., 2021; Dong *et al*., 2022; Nascimento *et al*., 2022). However, comparatively little is known about the enzymatic control and genetic basis of MeSA biosynthesis in roots and how this process shapes belowground interactions.

Tomato (*Solanum lycopersicum*) represents a well‐established model for studying MeSA biosynthesis and function (Martina *et al*., 2021). MeSA has been detected in tomato leaves and fruits, in which modulation of *SlSAMT* expression influences MeSA accumulation and plant defense responses to herbivores and pathogens (Ament *et al*., 2010; Tieman *et al*., 2010; Rambla *et al*., 2014). In fruits, elevated MeSA levels are associated with reduced consumer preference due to their wintergreen‐like aroma (Tieman *et al*., 2017; Lu *et al*., 2025). Accordingly, recent studies leveraging the tomato Varitome collection, which captures extensive genetic diversity across wild, semi‐domesticated, and cultivated accessions, have revealed substantial natural variation in fruit MeSA levels and identified associated genetic loci through genome‐wide association study (GWAS), providing potential targets for improving fruit quality (Tieman *et al*., 2017; Razifard *et al*., 2020; Frick *et al*., 2023; Sapkota *et al*., 2023; Lu *et al*., 2025). In addition, MeSA has also been detected in tomato roots (Murungi *et al*., 2018), and synthetic MeSA has been demonstrated to be used by root‐knot nematode (RKN, *Meloidogyne incognita*) juveniles as a chemoattractant (Kihika *et al*., 2017; Murungi *et al*., 2018). However, the genetic and biochemical basis of MeSA biosynthesis in roots, as well as its biological significance in belowground plant–pathogen interactions, remain largely unresolved. Here, we combine population‐scale metabolite profiling, genetic mapping, biochemical characterization, and functional analyses to investigate the genetic control and biological significance of MeSA biosynthesis in tomato roots, with a particular focus on its role in plant–nematode interactions.

## Materials and Methods

### Plant growth, bacterium, and chemical sources

For GWAS, we analyzed 167 accessions of tomato from the Varitome diversity panel (Sapkota *et al*., 2023). These included 28 accessions of wild *Solanum pimpinellifolium* L. (SP), 121 accessions of semi‐domesticated *Solanum lycopersicum* var. *cerasiforme* (SLC), and 18 accessions of ancestral landrace *Solanum lycopersicum* var. *lycopersicum* (SLL). Also investigated were two types of *SlSAMT* transgenic tomato, including one overexpression (OE) line and three antisense (AS) lines, all in the wild‐type (WT) M82 background (Tieman *et al*., 2010). Tomato plants were grown in one‐gallon pots containing 3B Mix (Fafard, Bradenton, FL, USA) and fertilized with Osmocote (Scotts, Marysville, OH, USA) in a glasshouse at a 16 h : 8 h, 26°C : 22°C, light : dark cycle (day : night). *Escherichia coli* strain BL21 (DE3) CodonPlus (Stratagene, La Jolla, CA, USA) was used for expressing the recombinant protein. The chemicals used in this study were purchased from Sigma‐Aldrich (St. Louis, MO, USA).

### Metabolic profiling of tomato roots

Volatile metabolites were profiled from 6‐wk‐old tomato roots using a solid‐phase microextraction (SPME)‐based method as previously described (Wang *et al*., 2026) for direct quantification analysis of volatiles in plant tissues. Approximately 100 mg fresh root tissue was ground in liquid nitrogen and dissolved in 20% NaCl supplemented with 0.001% 1‐octanol as an internal standard. Volatile compounds were collected by SPME for 30 min at 50°C to enhance volatile release and improve detection sensitivity before gas chromatography mass spectrometry (GC‐MS) analysis and subsequently analyzed by GC‐MS (Shimadzu) following the previously described program (Wang *et al*., 2026). Compounds were identified using the Wiley 12th edition/NIST 2020 mass spectral library and confirmed with synthetic standards. Three biological replicates were analyzed per sample, and relative metabolite abundance was calculated by normalizing analyte peak areas to the internal standard.

### Variant calling and GWAS

Short‐read whole‐genome sequencing data for the Varitome tomato accessions were retrieved from the NCBI Sequence Read Archive (BioProject PRJNA454805) and aligned to the *Solanum lycopersicum* L. reference genome SL4.0. Variant calling for single‐nucleotide polymorphisms (SNPs), small insertions and deletions (INDELs), and structural variants (SVs) was performed using previously established pipelines (Pereira *et al*., 2021; Sapkota *et al*., 2023). Briefly, raw reads were quality filtered, aligned, and jointly processed to generate genome‐wide variant datasets for each variant class.

Variants were filtered to retain only biallelic sites with < 5% missing data, minor allele frequency (MAF) ≥ 5%, and mapping quality ≥ 30 using BCFtools (Danecek *et al*., 2021). After filtering, 659 002 high‐quality INDELs were retained. To reduce marker redundancy and computational burden, SNPs were further thinned to one SNP per kilobase using vcftools (Danecek *et al*., 2011), resulting in a final dataset of 609 034 SNPs. Filtered SNPs and INDELs were converted to HapMap format using tassel v.5.0 (Bradbury *et al*., 2007). A total of 27 477 high‐confidence SVs from the final merged SV dataset were encoded as a binary presence/absence matrix and treated as pseudo‐SNPs, with the reference allele representing absence and the alternate allele representing presence of the SV. These SVs were likewise converted into HapMap format for downstream analyses.

GWAS analyses were conducted using the Bayesian‐information and Linkage‐disequilibrium Iteratively Nested Keyway (BLINK) model implemented in the GAPIT R package, following established procedures (Frick *et al*., 2023). GWAS were performed separately for SNPs, INDELs, and SVs, as recommended for multivariant datasets and as applied in prior tomato studies (Frick *et al*., 2023; Sapkota *et al*., 2023; Kayikci *et al*., 2025). For all analyses, variants with MAF < 5% were excluded, and false discovery rate (FDR) correction was applied to account for multiple testing. Associations were considered significant at an FDR‐adjusted *P* < 0.05. These analyses were performed on the Morgan Compute Cluster at the University of Kentucky (UKY) (https://www.ccs.uky.edu/).

Quantitative trait loci (QTL) intervals were defined by extending 200‐kb upstream and downstream of the lead variant, corresponding to the genome‐wide extent of linkage disequilibrium (LD) decay estimated for the Varitome population. Genome‐wide LD decay was estimated from the filtered SNPs using PopLDdecay (Zhang *et al*., 2018), and the genomic distance at which mean pairwise LD decayed to *r* ^2^ = 0.1 was used to define the 200‐kb QTL window. Pairwise LD between lead GWAS variants and neighboring markers was quantified as the squared Pearson correlation coefficient (*r* ^2^) using allele dosage‐coded genotypes (0, 1, 2). Genotypes were numerically encoded for each marker based on the observed reference and alternate alleles, and pairwise *r* ^2^ values were calculated between each lead variant and all variants within the target region using custom R scripts. Haplotype analyses based on SNP and INDEL variation were performed as previously described (Pereira *et al*., 2021).

### Phylogenetic analysis

The tomato (*Solanum lycopersicum* cv Heinz 1706) genome (SL4.0, ITAG4.0 annotation) was obtained from public databases (Phytozome). Putative SABATH family members were identified using a hidden Markov model‐based search against the Methyltransf_7 profile (PF03492) with an E‐value threshold of < 1 × 10^−5^ (Finn *et al*., 2016). Redundant sequences showing 100% identity were removed. Multiple sequence alignments were generated using mafft (v.7.407) with the localpair algorithm and 1000 iterative refinements (Katoh & Standley, 2013). The best‐fitting amino acid substitution model was determined using modeltest‐ng (v.0.1.7) with default parameters. Maximum‐likelihood phylogenetic tree was constructed using raxml‐ng (v.1.1.0) under the selected model with 1000 bootstrap replicates. Phylogenetic tree was visualized and annotated using itol (v.6.9.1) (Letunic & Bork, 2024). All bioinformatic analyses were conducted on the ISAAC‐NG high‐performance computing cluster at the University of Tennessee (https://oit.utk.edu/hpsc/isaac‐open‐enclave‐new‐kpb).

### Gene expression data retrieval and analysis

Gene expression data for tomato (*S. lycopersicum* cv Heinz) across different tissues were retrieved from the Tomato eFP Browser hosted at the BAR database (http://bar.utoronto.ca/) (Fucile *et al*., 2011) using gene ID‐based queries. Expression values were obtained as reads per kilobase per million mapped reads (RPKM) and used for comparative analysis of tissue‐specific expression patterns. Transcriptomic data from tomato roots infected with RKN were obtained from a published study (Ozdemir *et al*., 2024), in which RNA‐seq analyses were performed on RKN‐infested and control roots. The authors provided raw read count data for individual genes across treatments and corresponding controls. These data were reanalyzed in R to calculate differential gene expression, with fold changes computed relative to control samples and expressed as log_2_ (fold change) values. Heatmaps were generated in R to visualize transcriptional responses of selected genes to RKN infection.

### Cloning full‐length cDNA of tomato *SABATH* members

Total RNA was extracted using the RNeasy Plant Mini Kit (Qiagen, Valencia, CA, USA), including on‐column DNase treatment to remove genomic DNA. First‐strand cDNA was synthesized from 1.5 μg total RNA using the First‐strand cDNA Synthesis Kit (Amersham Biosciences, Piscataway, NJ, USA) following the manufacturer's instructions (Chen *et al*., 2003). Using specific forward and reverse primers (Supporting Information Table S1), the PCR program was conducted to amplify the full‐length cDNA sequences of target genes as follows: 98°C for 2 min, followed by 35 cycles at 98°C for 30 s, 57°C for 30 s, and 72°C for 1 min, and a final extension at 72°C for 10 min. The PCR products were separated on a 1.0% agarose gel. The target band was excised from the gel, purified using the QIAquick Gel Extraction kit (Qiagen), and cloned into the pET‐32a vector (Invitrogen), and confirmed by Sanger sequencing (Eurofins, Lancaster, PA, USA).

### Recombinant protein expression and purification

Tomato SABATH constructs were transformed into *E. coli* BL21 (DE3) CodonPlus (Stratagene) and recombinant protein expression was induced with 500 μM isopropyl *β*‐D‐1‐thiogalactopyranoside (IPTG) for 18 h at 25°C. Cells were lysed by sonication, and His‐tagged proteins were purified using nickel‐nitrilotriacetic acid (Ni‐NTA) agarose (Invitrogen). Protein purity was assessed by sodium dodecyl sulfate–polyacrylamide gel electrophoresis (SDS‐PAGE), and concentrations were determined using the Bradford assay (Bradford, 1976).

### Methyltransferase enzyme assays *in vitro*

Methyltransferase activity was assayed using both radiochemical and nonradiochemical systems. For radiochemical assays, reactions were performed in a 50 μl volume containing 50 mM Tris–HCl (pH 7.5–7.8), 1 mM substrate, purified protein, and S‐[methyl‐^14^C]‐adenosyl‐L‐methionine (^14^C‐SAM; 51.4 mCi mmol^−1^; PerkinElmer, Boston, MA, USA). Reactions were incubated at room temperature for 30 min and terminated by extraction with 150 μl ethyl acetate. Following centrifugation, radioactivity in the organic phase was quantified by liquid scintillation counting (Beckman Coulter, Fullerton, CA, USA) and taken as a measure of methyl ester formation (D'Auria *et al*., 2002). Data were presented as mean ± SE of three replicate enzyme assays. For nonradiochemical assays, unlabeled SAM was used as the methyl donor. Volatile reaction products were collected by SPME and analyzed by GC‐MS. Synthetic standard chemicals were analyzed by GC‐MS as described previously (Zhao *et al*., 2008).

### Root‐knot nematode infection assays and resistance evaluation

Nematode infection bioassays were performed to assess the effects of altered root MeSA production on tomato susceptibility to RKN (*Meloidogyne incognita* (Kofoid and White) Chitwood). Ten days after seed germination, each tomato seedling's root was inoculated with *c*. 1 ml of inoculum, which contained *c*. 400 freshly hatched second‐stage juveniles (J2). Plants were grown at 70% relative humidity and a 16 h : 8 h, 26°C : 22°C, light : dark photoperiod. Six weeks post inoculation, infected root samples were harvested and washed to remove soil. Galls were counted under a dissecting microscope (Fisher Scientific, Waltham, MA, USA). Eggs were extracted from infected roots using a 10% bleach solution for 4 min and were resuspended in water and quantified by examining serial dilutions under a dissecting microscope. Gall per root system and egg numbers per gram root tissues were used as indices of plant susceptibility. Experiments were conducted with 15 replicated plants per genotype.

### Statistical analysis

Root volatile measurements were obtained from three biological replicates per accession. For each volatile compound, replicate measurements were summarized as accession‐level least‐squares means (LS‐means) using linear models implemented in R. Specifically, LS‐means were estimated using the emmeans package (Lenth & Lenth, 2018) and used for downstream GWAS analyses. For group comparisons, LS‐means were analyzed using one‐way ANOVA, followed by Tukey's HSD test for multiple comparisons. Statistical significance thresholds are specified in the corresponding figure legends and table footnotes. For accessions in which MeSA was not detected, the phenotype was not treated as missing. Instead, these samples were considered to represent extremely low‐abundance MeSA phenotypes below the GC‐MS detection threshold and were assigned a minimal non‐zero value of 1 × 10^−21^ for downstream statistical analysis. Retaining these accessions preserved the full phenotypic range of root MeSA variation across the Varitome panel and avoided biasing GWAS by excluding genotypes with very low MeSA accumulation.

## Results

### Domestication‐associated reduction of MeSA levels in tomato roots

**Fig. 1:**
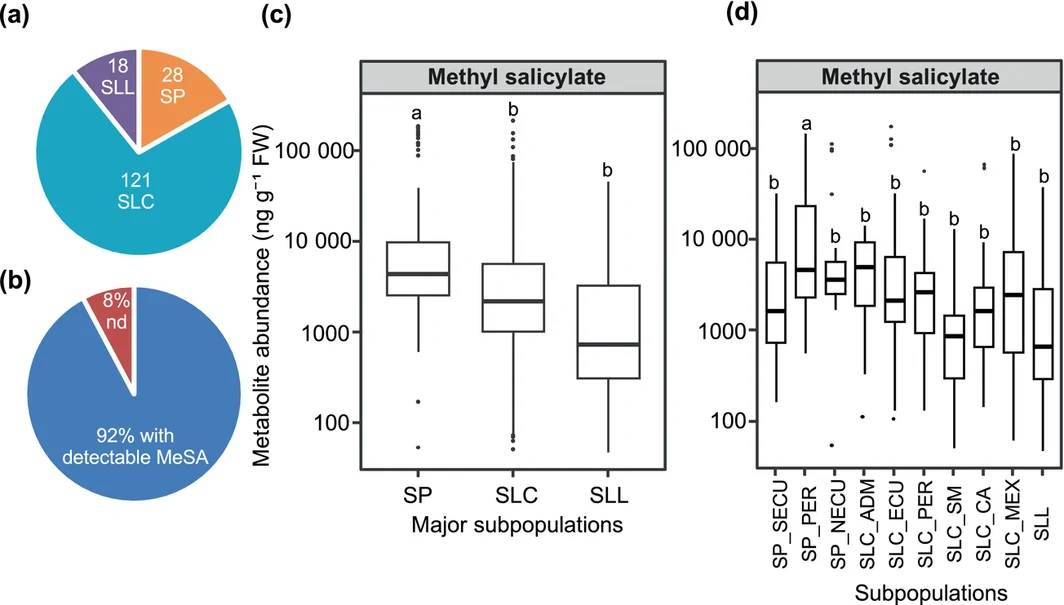
Root volatile profiling reveals domestication‐associated variation in methyl salicylate (MeSA) accumulation in tomato. (a) Composition of the tomato Varitome collection analyzed, comprising wild (*Solanum pimpinellifolium*, SP), semicultivated (*Solanum lycopersicum* var. *cerasiforme*, SLC), and cultivated (*Solanum lycopersicum* var. *lycopersicum*, SLL) accessions. (b) Percentage of accessions with detectable or nondetectable (nd) MeSA from roots across the tomato population. (c) Root MeSA levels in tomato accessions grouped by major subpopulations. (d) Root MeSA levels in tomato accessions grouped by subpopulations. SP_SECU, SP from southern Ecuador; SP_PER, SP from Peru; SP_NECU, SP from northern Ecuador; SLC_ADM, SLC admixture; SLC_ECU, SLC from Ecuador; SLC_PER, SLC from Peru; SLC_SM, SLC from San Martin; SLC_CA, SLC from Central America; SLC_MEX, SLC from Mexico; SLL, cultivated tomato accessions in the Varitome collection. Root MeSA level for each accession was determined from three independent biological replicates. For the boxplots in (c) and (d), the horizontal line within each box represents the median, the lower and upper limits of the box represent the first and third quartiles, respectively, the whiskers extend to the most extreme values within 1.5 times the interquartile range, and points beyond the whiskers represent outliers. Different letters indicate significant differences among subpopulations (adjusted *P* < 0.05).

To characterize natural variation in root volatiles across tomato populations, we profiled root volatiles in 167 accessions spanning three groups: 28 wild (*S. pimpinellifolium*; SP) accessions, 121 semi‐domesticated (*S. lycopersicum* var. *cerasiforme*; SLC) accessions, and 18 cultivated (*S. lycopersicum* var. *lycopersicum*; SLL) accessions (Sapkota *et al*., 2023) (Fig. 1a). MeSA was detected in 154 accessions (92% of the entire tomato population), including 27 of 28 SP accessions, 109 of 121 SLC accessions, and all 18 SLL accessions, making it the broadly conserved volatile in tomato roots (Fig. 1b). Across the population, MeSA was the dominant compound in nearly all genotypes (Fig. S1). MeSA levels displayed a clear domestication‐associated gradient: wild SP accessions accumulated significantly higher MeSA concentrations than semidomesticated accessions, which in turn showed higher levels than the cultivated ancestral accessions (Fig. 1c). Within the wild SP, accessions originating from Peru (SP_PER) displayed particularly high MeSA accumulation (Fig. 1d). Together, these results indicate that while MeSA production is broadly conserved across tomato lineages, its abundance shows a progressive reduction associated with domestication.

### GWAS identifies multiple loci for root MeSA, including a reproducible signal at SlSAMT region on Chromosome 9

**Fig. 2:**
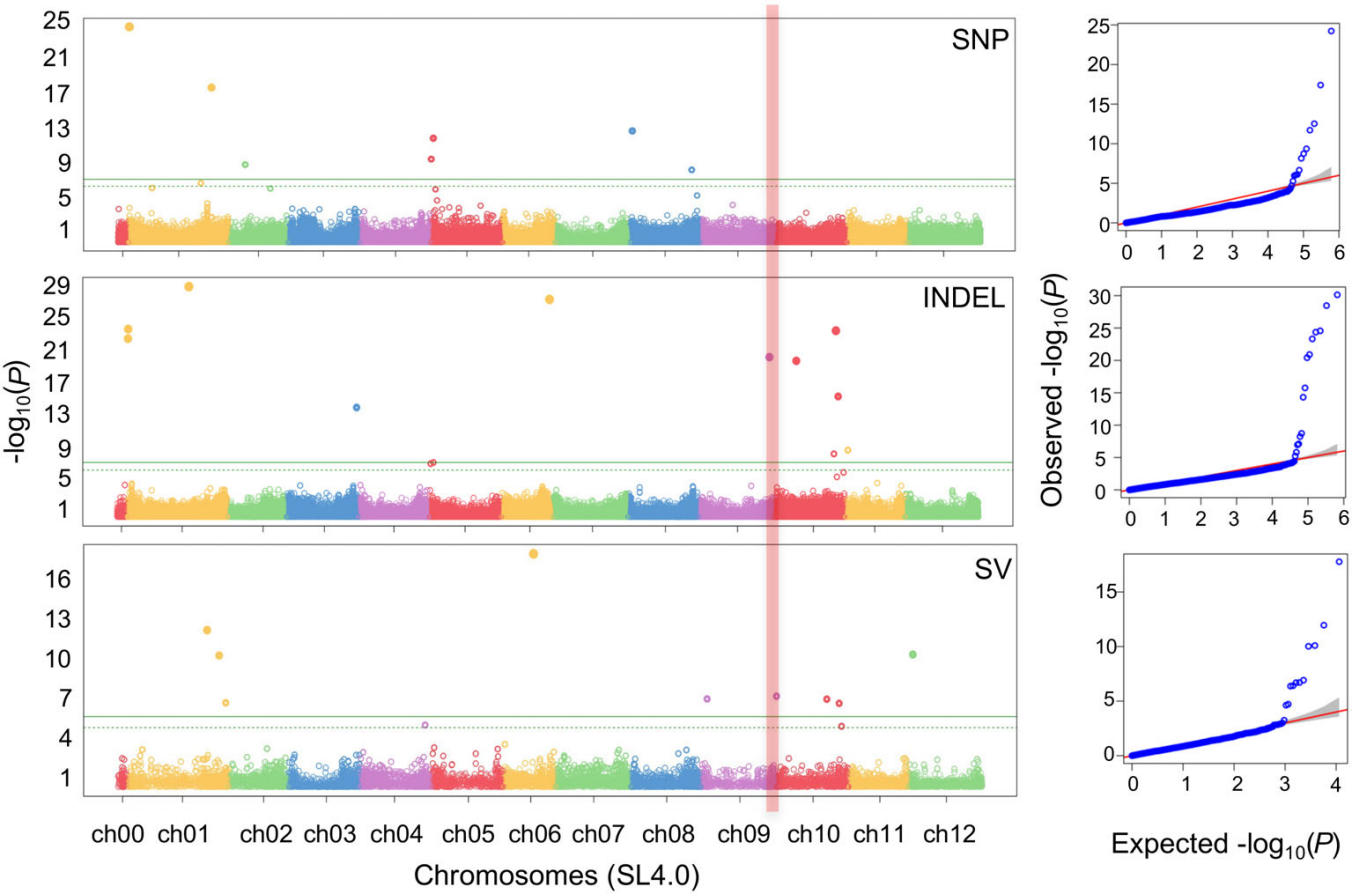
Genome‐wide association analysis identifies a major locus controlling root methyl salicylate (MeSA) accumulation in tomato. Left, Manhattan plots showing genome‐wide association analysis results for root MeSA levels in the tomato Varitome collection based on single‐nucleotide polymorphisms, insertions/deletions, and structural variants. The *x*‐axis represents genomic positions across the 12 tomato chromosomes, and the *y*‐axis shows –log_10_(*P*) values for variant‐trait associations. Right, Quantile–quantile (QQ) plots indicate minimal inflation of test statistics, supporting appropriate model fitting. Significant peaks represent loci associated with variation in root MeSA accumulation.

To uncover genetic factors underlying population‐wide MeSA variation, we performed a GWAS using root MeSA levels from the 167 accessions, combined with genome‐wide SNP, INDEL, and SV datasets. Although MeSA was classified as undetected in 13 accessions, these observations were retained in GWAS because they represented biologically meaningful low‐abundance phenotypes rather than missing data. These accessions were assigned a minimal nonzero value of 1 × 10^−21^ for downstream analysis, thereby preserving the full range of natural variation in root MeSA accumulation. GWAS was performed separately for each variant class using the BLINK model. Quantile–quantile plots indicated appropriate control of population structure and relatedness (Fig. 2). Across all variant types, GWAS identified 27 significant marker‐trait associations (designated *MeSA1.1*–*MeSA12.1*; Table S2) distributed across the genome, which resolved into 24 distinct QTLs after consolidating signals within ±200 kb LD intervals (Fig. S2). This was consistent with a polygenic architecture for root MeSA accumulation. Notably, several of these QTLs overlapped with members of the SABATH methyltransferase family (Table S3), motivating a focused evaluation of methyltransferase candidates in the following section. This enrichment of associations with genes with known roles in salicylate and benzenoid methylation suggested a direct genetic connection between SABATH family variation and natural diversity in root MeSA levels.

Among all detected loci, a prominent signal on Chromosome 9 was repeatedly supported across variant types (*MeSA9.2* and *MeSA9.3*; Fig. S3). The lead associations included an INDEL at 64.45 Mb (*MeSA9.2*; ISL4.0ch09_64449339; percentage of phenotypic variance explained (PVE) = 11.95%) and a 987‐bp deletion SV at 67.99 Mb (*MeSA9.3*; VSL4.0CH09_67992261; PVE = 2.94%). These lead variants flank the *SlSABATH* gene cluster on Chromosome 9 (*Solyc09g091530*, *Solyc09g091540*, and *Solyc09g091550*), which includes the cloned *SlSAMT* (*Solyc09g091550*; SL4.0ch09: 66.901–66.904 Mb) (Tieman *et al*., 2010) (Table S2). Relative to *SlSAMT*, the INDEL lead variant lies *c*. 2.45 Mb upstream and the SV lead variant *c*. 1.09‐Mb downstream, placing the cluster within the broader associated interval. Local LD analysis further supported the potential association between the chromosome 9 signal and the SlSAMT region: two INDELs located *c*. 154 kb upstream of *SlSAMT* showed high LD (*r* ^2^ > 0.5) with the SV lead variant (Figs S2, S3). Notably, an independent GWAS of fruit MeSA also detected an association near SlSAMT (SNP at SL4.0ch09: 67197164; *c*. 293 kb downstream of SlSAMT) (Sapkota *et al*., 2023), consistent with this region contributing to MeSA variation across plant tissues. Haplotype analysis across the *SlSAMT* locus identified two major haplotypes, including a wild‐enriched haplotype prevalent in *S. pimpinellifolium* and a cultivated‐enriched haplotype predominant in SLC (Fig. S4). These haplotypes were associated with significantly different root MeSA levels (ANOVA, *P* = 0.0142), with wild‐enriched haplotype exhibiting higher MeSA than cultivated‐enriched haplotype (Fig. S4). This directional difference is consistent with the domestication‐associated gradient observed at the phenotypic level, supporting natural allelic variation at/near the chromosome 9 *SlSAMT* region contributing to root MeSA variation.

### *Solyc09g091550* is the only gene in the three tandem paralogs at the *MeSA9.3* locus that has SAMT activity

**Fig. 3:**
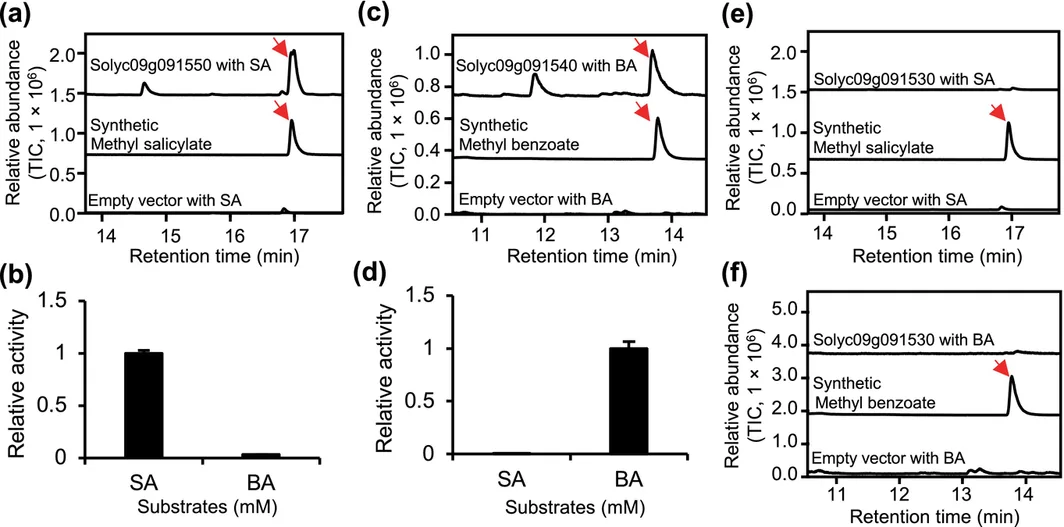
Biochemical characterization of tomato SABATH enzymes' catalytic activities. (a) Gas chromatography–mass spectrometry (GC‐MS) showing the reaction product (indicated by red arrow) catalyzed by Solyc09g091550 (SlSAMT) using salicylic acid (SA) as the substrate. (b) Relative catalytic activity of recombinant SlSAMT protein with benzoic acid (BA) or SA as substrates; its catalytic activity with SA is arbitrarily set at 1.0. (c) GC‐MS showing the reaction product (indicated by red arrow) catalyzed by Solyc09g091540 (SlBAMT1) using BA as the substrate. (d) Relative catalytic activity of recombinant SlBAMT1 protein with BA or SA as substrates; its catalytic activity with BA is arbitrarily set at 1.0. (e, f) GC‐MS showing the reaction product catalyzed by Solyc09g091530 using BA or SA as substrates. Synthetic standards are indicated by red arrows. Data represent mean ± SE of three independent enzyme assays.

Within the *MeSA9.3* interval, a tandem cluster of three SABATH paralogs – Solyc09g091530, *Solyc09g091540*, and *Solyc09g091550 – represents* the strongest candidate region for MeSA biosynthesis on Chromosome 9. While *Solyc09g091550* had been previously demonstrated to encode SAMT (Tieman *et al*., 2010), the catalytic activity of the other two paralogs was unknown. To determine whether they also encode SAMT, full‐length cDNAs for these two genes together with *Solyc09g091550* as a positive control were all cloned and expressed in *E. coli* to produce recombinant proteins. Recombinant proteins were then tested for catalytic activity using SA and benzoic acid (BA) as specific substrates; the latter was included due to its structural similarity to SA. Enzymatic assays showed that only Solyc09g091550 (SlSAMT) catalyzed the methylation of SA to produce MeSA with high efficiency and displayed only minimal activity toward BA (Fig. 3a,b), establishing it as the SA‐specific methyltransferase among the three. By contrast, Solyc09g091540 (SlBAMT1) efficiently methylated BA to methyl benzoate (MeBA) and showed negligible activity toward SA (Fig. 3c,d), whereas Solyc09g091530 exhibited no detectable activity toward either substrate (Fig. 3e,f).

### Systematic SABATH family analysis supports SlSAMT as the primary SA‐methylating enzyme in tomato

To determine whether additional SABATH members in tomato contribute to the biosynthesis of MeSA, we performed a systematic analysis of the SABATH family in tomato. The tomato genome contains 18 *SABATH* genes, encoding proteins of 276–410 amino acids (Table S3). Five of these enzymes have been previously characterized, including SlSAMT, SlJMT (jasmonic acid methyltransferase), SlIAMTs (indole‐3‐acetic acid methyltransferases), and SlPAAMT (phenylacetic acid methyltransferase) (Tieman *et al*., 2010; Wang *et al*., 2025).

**Fig. 4:**
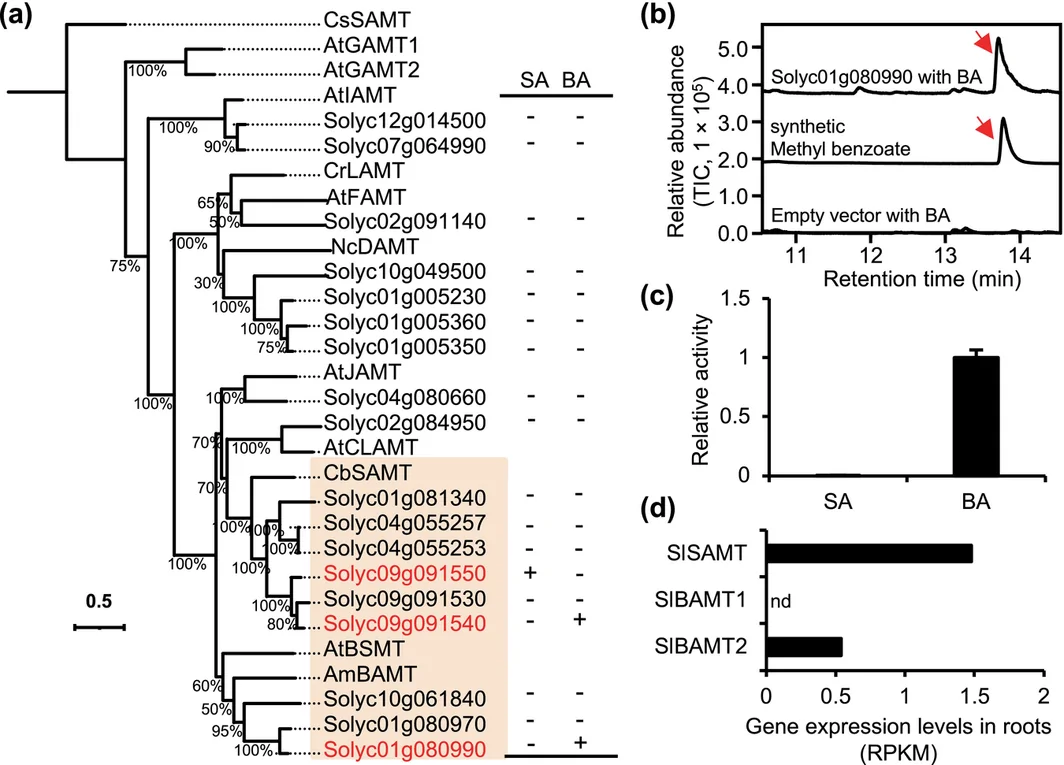
Phylogenetic relationships and biochemical activities of the tomato SABATH methyltransferase family. (a) Maximum‐likelihood phylogeny (left) of tomato SABATH methyltransferases together with previously characterized plant SABATH proteins. The tomato genome contains 18 SABATH members. SAMT‐like and BAMT/BSMT‐like clades are highlighted in orange. The scale bar represents 0.5 amino acid substitutions per site, and bootstrap values indicate node support. Previously characterized SABATHs from other plant species are shown as: CbSAMT, salicylic acid methyltransferase from *Clarkia breweri* (Ross *et al*., 1999); AmBAMT, benzoic acid methyltransferase from *Antirrhinum majus* (Dudareva *et al*., 2000); AtJAMT, jasmonic acid methyltransferase (Seo *et al*., 2001), AtBSMT1, benzoic acid/salicylic acid methyltransferase (Chen *et al*., 2003), AtIAMT1, indole‐3‐acetic acid methyltransferase (Qin *et al*., 2005), AtFAMT, farnesoic acid methyltransferase (Yang *et al*., 2006), AtGAMTs, gibberellic acid methyltransferases (Varbanova *et al*., 2007), and AtCLAMT, carlactonoic acid methyltransferase (Wakabayashi *et al*., 2021) from *Arabidopsis thaliana*; CsLAMT, loganic acid methyltransferase from *Catharanthus roseus* (Murata *et al*., 2008); NcDAMT, decanoic acid methyltransferase from *Nymphaea colorata* (Zhang *et al*., 2020); CsSAMT from the liverwort *Conocephalum salebrosum* (Zhang *et al*., 2019), included as a distantly related, nonflowering‐plant SABATH to provide a broader phylogenetic context. The table (right) displays the catalytic activities of tomato SABATH proteins using benzoic acid (BA) or salicylic acid (SA) as substrates, where ‘+’ and ‘−’ indicate detectable and undetectable activity, respectively. (b) Gas chromatography–mass spectrometry showing the reaction product (indicated by red arrow) catalyzed by Solyc01g080990 (SlBAMT2) using BA as the substrate. (c) Relative catalytic activity of recombinant SlBAMT2 protein with BA or SA as substrates; its catalytic activity with BA is arbitrarily set at 1.0. Data represent mean ± SE of three independent assays. (d) Gene expression levels of *SlSAMT* and *SlBAMTs* in tomato roots. ‘nd’ indicates no detectable expression. Expression values are shown as reads per kilobase of transcript per million mapped reads (RPKM) and were obtained from the BAR database.

To place these biochemical results in an evolutionary context and to assess other potential contributors, we next examined the phylogeny of tomato SAMT‐like and BAMT/BSMT‐like SABATHs. The latter often showing dual activity with both BA and SA and is also referred to as SA/benzoic acid methyltransferases (BSMTs) (Dubs *et al*., 2022). Phylogenetic analysis resolved the tomato SlSAMT and SAMT‐like genes, clustered with CbSAMT from *Clarkia breweri*, and BAMT/BSMT‐like genes, clustered with AtBSMT from *Arabidopsis thaliana* and AmBAMT from *Antirrhinum majus*, into two independent clusters (Fig. 4a). A well‐supported SAMT‐like clade (bootstrap = 100%) that includes CbSAMT from *Clarkia breweri* (Ross *et al*., 1999) further subdivided into two strongly supported subclades (each bootstrap = 100%): the Solyc09g091530–550 cluster including SlSAMT, and a sister subclade containing three additional SAMT‐like paralogs. A separate, weakly supported BAMT/BSMT‐like clade (bootstrap = 60%) included AtBSMT from *A. thaliana* (Chen *et al*., 2003), AmBAMT from *A. majus* (Dudareva *et al*., 2000), and three additional tomato BAMT/BSMT‐like members. Because these two lineages both include several paralogs beyond the top‐QTL cluster, we extended enzymatic assays to these remaining candidates. Solyc01g080990 (SlBAMT2) efficiently catalyzed BA methylation but showed no activity toward SA (Fig. 4b,c), confirming its identity as a BA methyltransferase. Other SAMT‐like and BAMT/BSMT‐like SABATH proteins or other clades SABATH proteins exhibited no detectable activity with either SA or BA under our assay conditions (Fig. 4a). Collectively, these biochemical data demonstrate that SlSAMT is the primary SA‐specific methyltransferase within the SAMT‐like lineage, with the gene expression in tomato roots (Fig. 4d). SlSAMT has been linked previously to fruit MeSA production (Tieman *et al*., 2010; Sapkota *et al*., 2023), yet its effect was smaller than that of the major fruit MeSA loci such as nonsmoky glucosyl transferase (1) and salicylic acid methyl esterase 1 (*SlMES*) (Sapkota *et al*., 2023). Therefore, our results extend this relationship to roots by showing that SlSAMT is the primary SABATH enzyme with SA‐methylating activity in both root and fruit MeSA production.

### Levels of MeSA in roots are correlated with altered expression of *SlSAMT* in transgenic tomato plants

**Fig. 5:**
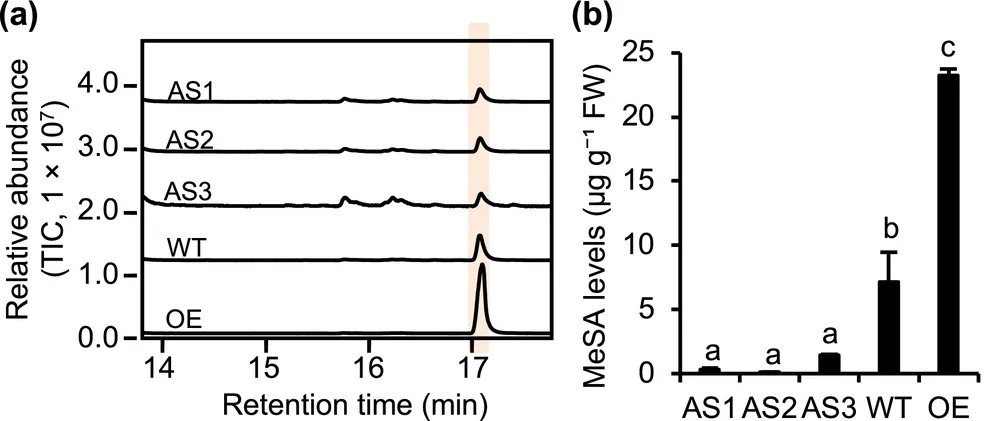
Methyl salicylate accumulation in transgenic tomato roots. (a) Gas chromatography–mass spectrometry profiles of volatile metabolites from transgenic tomato roots, with methyl salicylate (MeSA) highlighted by the orange bar. (b) Quantification of MeSA levels in transgenic tomato roots. WT represents the wild‐type tomato cultivar M82. Transgenic lines, generated in the M82 background, include one *SlSAMT* overexpression line (OE) and three *SlSAMT* antisense lines (AS1–AS3). Data represent mean ± SE of three independent biological replicates. Different letters indicate significant differences among genotypes (adjusted *P* < 0.05).

To determine whether SlSAMT controls tomato root MeSA production *in vivo*, we profiled MeSA levels in previously generated *SlSAMT*‐OE and ‐AS lines, which used the tomato cultivar M82 (a processing‐type variety) as the background (Tieman *et al*., 2010). GC‐MS chromatograms confirmed that MeSA is the major root volatile across all genotypes; however, peak intensities differed markedly among lines. The OE line exhibited a noticeably higher MeSA peak than WT, whereas all three AS lines showed greatly diminished peaks (Fig. 5a). Quantitative analyses further revealed that compared with WT (7.12 μg g^−1^ FW), root MeSA levels in the OE line were dramatically elevated by approximately threefold (23.22 μg g^−1^ FW), whereas three AS lines (AS1, 2, 3) showed strongly reduced MeSA levels of *c*. 0.02–0.2‐fold (0.12–1.48 μg g^−1^ FW) (Fig. 5b). These results demonstrate that SlSAMT is the primary enzyme regulating MeSA accumulation in tomato roots, functioning specifically at the SA→MeSA conversion step *in planta*.

### SlSAMT‐mediated MeSA biosynthesis modulates root susceptibility to root‐knot nematodes

**Fig. 6:**
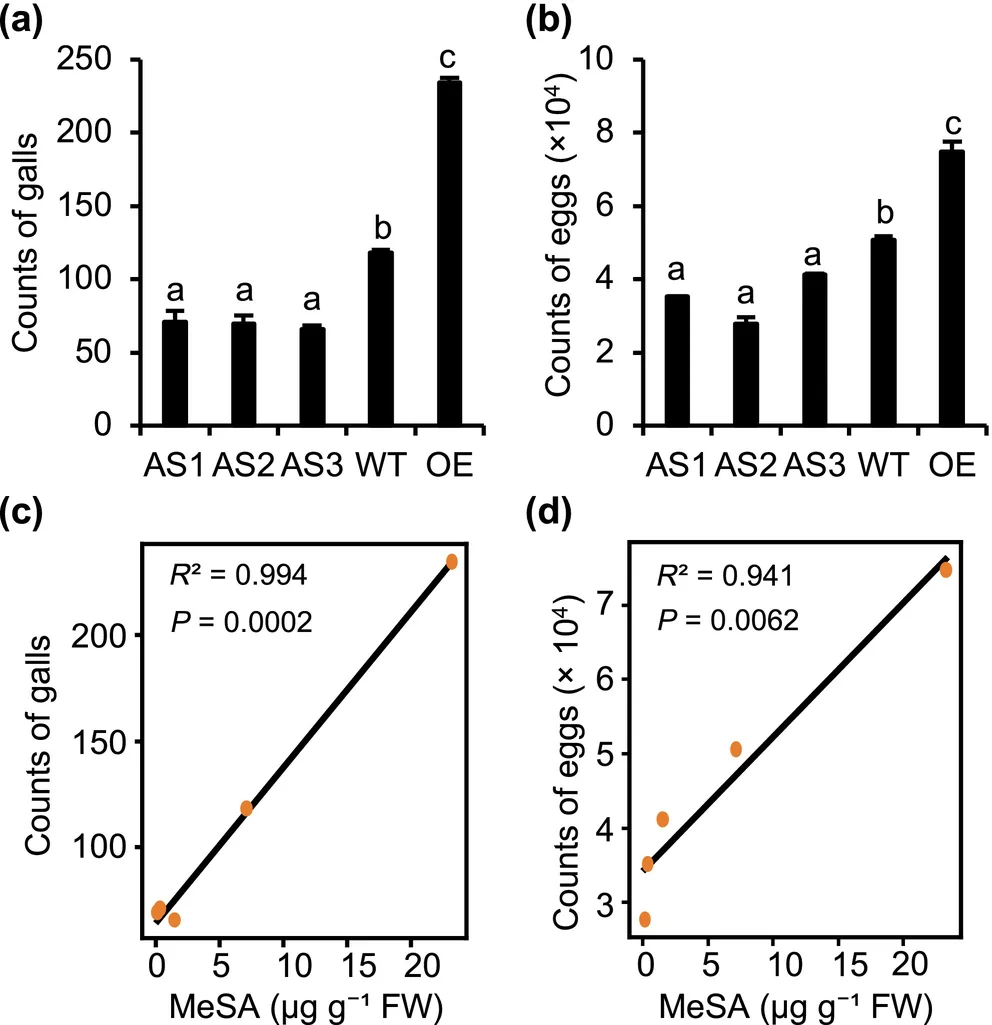
Root‐knot nematode (RKN) performance in SlSAMT transgenic tomato roots. RKN infection assays were performed using tomato plants in the M82 background, including one SlSAMT overexpression line (OE), three SlSAMT antisense lines (AS1–AS3), and wild‐type (WT) M82 as the control. (a) Number of galls per root system and (b) number of eggs per gram root tissues, used as indices of host susceptibility. Bars represent means ± SE from 15 plants per genotype. Different letters indicate statistically significant differences among genotypes (adjusted *P* < 0.01). (c, d) Linear regression lines show the relationships between root methyl salicylate levels and gall number per root system or egg number per gram of root tissues in tomato plants infected with RKN. Values indicate the coefficient of determination (*R* ^2^) and adjusted *P* values.

Synthetic MeSA was previously shown to attract *M. incognita* (Murungi *et al*., 2018), yet the genetic determinants of MeSA production in tomato roots remained unknown. Therefore, we tested whether SlSAMT activity directly modulates plant susceptibility to nematode infection. To assess the biological significance of SlSAMT‐mediated MeSA biosynthesis in root defense, we evaluated the susceptibility of these lines to infection by the RKN *M. incognita*. Six weeks after inoculation, all genotypes developed galls and egg masses, but the severity varied strongly with *SlSAMT* expression level. The OE line exhibited the highest galls (234.67) and egg production (7.48 × 10^4^), approximately twofold and 1.5‐fold higher than WT, respectively (Fig. 6a,b). By contrast, AS lines displayed significantly fewer galls (*c*. 68.70) and egg (*c*. 3.48 × 10^4^) counts, averaging 0.6‐fold of the WT levels, indicating enhanced resistance (Fig. 6a,b). Across all genotypes, gall or egg numbers were strongly positively correlated with MeSA accumulation (Fig. 6c,d). Together, these findings demonstrate that SlSAMT‐driven MeSA biosynthesis modulates tomato root susceptibility to RKN infection. Elevated MeSA production compromises resistance, whereas reduced SlSAMT expression, and consequently lower MeSA levels, confers enhanced resistance, possibly through altered SA‐dependent defense signaling.

### Local SlSAMT induction and SA‐pathway reprogramming under nematode infection

Given that increased *SlSAMT* expression reduced tomato resistance to RKN, we examined how *SlSAMT* expression was affected by nematode infection. We analyzed recently published transcriptomes of *M. incognita*‐infected tomato roots at early (4 d post inoculation (dpi)) and late (11 dpi) stages (Ozdemir *et al*., 2024). Among all *SAMT*‐like and *BAMT/BSMT*‐like members, *SlSAMT* was the only gene consistently and strongly induced in both gall tissues and neighboring root segments (Fig. S5). *SlSAMT* transcript levels increased 2.8‐ to 3.8‐fold in galls relative to uninfected roots at both infection stages. A marked induction (*c*. 2.5‐fold) was also observed in tissues immediately surrounding galls at 4 dpi, indicating localized activation of MeSA biosynthesis during nematode establishment. Although *SlSAMT* expression declined slightly by 11 dpi, it remained elevated relative to controls, suggesting sustained involvement throughout gall development.

**Fig. 7:**
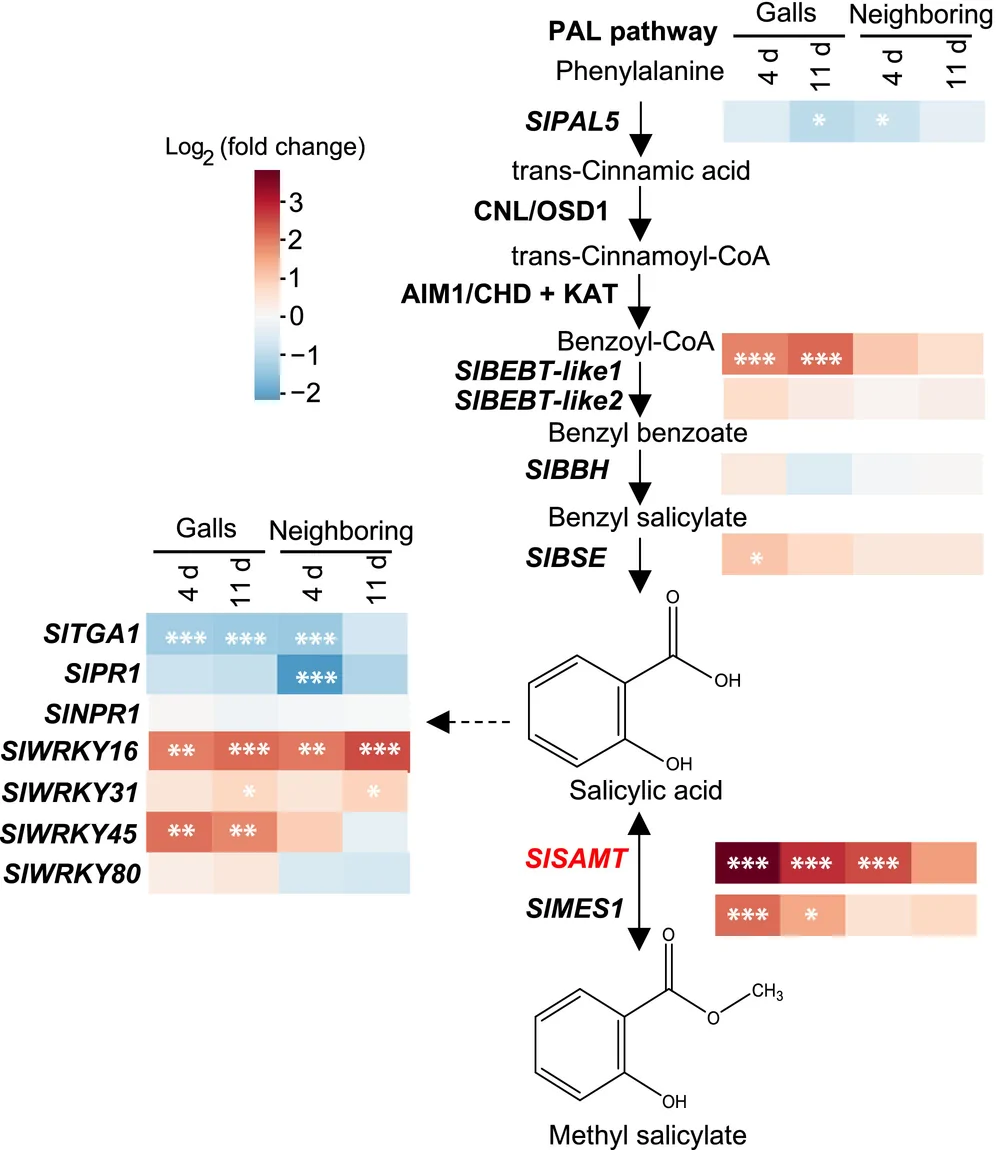
Expression patterns of selected salicylic acid (SA)‐related tomato genes during root‐knot nematode infection. The diagram summarizes the phenylalanine ammonia‐lyase (PAL)‐derived SA biosynthetic route, the SA‐methyl salicylate (MeSA) interconversion step, and selected genes of the SA signaling pathway (pointed by a dashed arrow). Heatmaps show log_2_ (fold change) expression of selected tomato genes in galls and neighboring root tissues at 4 d and 11 d post inoculation following *Meloidogyne incognita* infection. Fold changes were calculated relative to uninfected control roots. Asterisks indicate adjusted *P* values calculated using the Wald test implemented in the DESeq2 with Benjamini–Hochberg correction for multiple testing. The number of asterisks indicates the level of statistical significance based on adjusted *P* values: *, **, and *** correspond to adjusted *P* < 0.05, < 0.01, and < 0.001, respectively. Tomato gene names in italics indicate genes or homologs for which expression values were extracted (Supporting Information Table S4). Nonitalicized enzyme/module names indicate pathway steps for which tomato gene IDs were not assigned in this figure. Expression values were extracted from a published transcriptome dataset (Ozdemir *et al*., 2024). The color scale represents log_2_ (fold change) values. Sl, *Solanum lycopersicum*; PAL5, phenylalanine ammonia‐lyase 5A; CNL, cinnamoyl‐CoA ligases; OSD1, SA‐deficient gene 1; AIM1, abnormal inflorescence meristem 1; CHD, cinnamoyl‐CoA hydratase/dehydrogenase; KAT, 3‐ketoacyl‐CoA thiolase; BEBT, benzoyl‐CoA:benzyl alcohol benzoyltransferase; BBH, benzylbenzoate hydroxylase; BSE, benzylsalicylate esterase; SAMT, salicylic acid methyltransferase; MES1, salicylic acid methyl esterase 1; NPR1, nonexpressor of pathogenesis‐related gene 1; TGA1, TGA[C/T]G motif‐binding transcription factors 1; PR1, pathogenesis‐related protein 1; WRKYs, WRKY transcription factors.

Next, we examined the expression of selected tomato genes of the SA biosynthetic and signaling pathways (Table S4; Fig. 7) using the same transcriptome dataset. Recent studies have clarified the phenylalanine ammonia‐lyase (PAL)‐derived SA biosynthetic pathway by defining a route from benzoyl‐CoA to SA via benzyl benzoate and benzyl salicylate (Liu *et al*., 2025; Wang *et al*., 2025; Zhu *et al*., 2025). Within the PAL‐derived SA pathway (Fig. 7), *SlBEBT‐like1* (benzoyl‐CoA:benzyl alcohol benzoyltransferase‐like protein) showed clear induction in galls, while *SlBSE* (benzyl salicylate esterase) was induced mainly at 4 dpi (Wang *et al*., 2025). By contrast, the representative PAL gene *SlPAL5* was reduced in several samples (Guo & Wang, 2009; Zhang *et al*., 2023). The induction of *SlMES1*, a reported MeSA‐demethylating enzyme (Frick *et al*., 2023; Lu *et al*., 2025), was weaker and less consistent that *SlSAMT* upon RKN infection, suggesting increased biosynthesis of MeSA as an outcome of nematode infection. Among SA‐associated signaling and defense genes (Fig. 7), *SlTGA1* (TGA[C/T]G motif‐binding transcription factor 1) (Zheng *et al*., 2025) and *SlPR1* (pathogenesis‐related protein 1) (Chen *et al*., 2023) showed reduced expression patterns, while *SlNPR1* (nonexpressor of pathogenesis‐related gene 1) showed little change (Li *et al*., 2019). Several reported RKN‐related *WRKY* transcription factor genes showed distinct responses: *SlWRKY16, 31* (Kumar *et al*., 2023) and *SlWRKY45* (Chinnapandi *et al*., 2017; Huang *et al*., 2022) showed different degrees of induction, whereas *SlWRKY80* (Nie *et al*., 2023; Chen *et al*., 2024) showed no clear response to RKN infection.

## Discussion

### SlSAMT is the primary enzyme controlling MeSA biosynthesis in tomato roots

Recent evidence from a broad survey of methyltransferases indicates that many BAMT/BSMT enzymes exhibit dual substrate specificity toward SA and BA, raising the possibility that multiple enzymes could contribute to MeSA formation (Dubs *et al*., 2022). However, previous studies of tomato MeSA biosynthesis have largely focused on leaf and fruit tissues and did not exclude potential functional redundancy among paralogs (Ament *et al*., 2010; Tieman *et al*., 2010). The tomato genome encodes a diverse SABATH methyltransferase family, including multiple SAMT‐like and BAMT/BSMT‐like candidates distributed across distinct phylogenetic clades (Fig. 4). By systematically characterizing candidate enzymes and integrating biochemical assays with genetic evidence, our results demonstrate that SlSAMT is the primary SA‐methylating enzyme responsible for MeSA production in tomato roots (Figs 3, 4). This finding extends the role of SlSAMT beyond aerial tissues and establishes its conserved catalytic function across plant organs. Furthermore, through transgenic manipulation and phenotypic analyses, we show that modulation of *SlSAMT* expression directly alters MeSA accumulation and, consequently, root susceptibility to RKNs. Overexpression of *SlSAMT* increases MeSA levels and enhances susceptibility, whereas suppression of *SlSAMT* reduces MeSA accumulation and confers increased resistance (Figs 5, 6). Together, these results provide evidence for a mechanistic link between MeSA biosynthesis and plant–nematode interactions.

### Context‐dependent roles of MeSA in plant defense and belowground interactions

MeSA has been extensively characterized as a defense‐related signal in aerial tissues, functioning as a key mobile signal in SAR and as a volatile mediator of plant–plant and plant–insect interactions (Singewar *et al*., 2021; Gondor *et al*., 2022; Gong *et al*., 2023). In these contexts, MeSA is generally associated with enhanced defense capacity (Rowen *et al*., 2017; Ninkovic *et al*., 2021; Ali *et al*., 2023). However, previous studies have shown that synthetic MeSA can serve as a chemoattractant for RKN juveniles (Kihika *et al*., 2017; Murungi *et al*., 2018). Building upon these observations, our findings identify the genetic basis of root MeSA production and demonstrate that genetically altering MeSA accumulation through manipulation of *SlSAMT* expression leads to corresponding changes in tomato susceptibility to RKN (Fig. 6). Together, these findings reveal that a metabolite traditionally associated with defense can also mediate interactions that increase host vulnerability in belowground environments. A possible explanation is that in belowground systems, where volatile blends are comparatively simple (Fig. S1), MeSA may represent a dominant and conserved chemical cue (Fig. S1), increasing its reliability as a signal, and may have been evolutionarily co‐opted by RKNs for host location. Our study provides a mechanistic example of illustrating the dynamic interplay between plant chemical defense and parasite adaptation.

In addition, recent studies have identified tomato methyl esterases (SlMESs), particularly SlMES1, as key functional enzymes that convert MeSA back to SA, thereby reducing MeSA accumulation (Frick *et al*., 2023; Lu *et al*., 2025). This reversible regulation of SA‐MeSA balance underscores the importance of shaping functional outcomes of plant defense signaling. Consistently, transcriptomic analyses reveal substantial reprogramming of SA‐associated pathways during nematode infection, with strong induction of *SlSAMT* and heterogeneous regulation of other components of SA biosynthesis and signaling (Fig. 7). These findings indicate the complexity of tomato defense against nematodes mediated by the SA signaling pathway and MeSA‐associated susceptibility to nematodes and warrant further investigation.

### Domestication has reshaped root MeSA levels through quantitative regulation

Across the tomato Varitome population, MeSA represents the dominant root volatile despite the overall low abundance of root‐emitted compounds (Fig. S1). Notably, root‐derived MeSA levels exhibit a clear domestication‐associated gradient, decreasing from wild to semidomesticated and cultivated accessions (Fig. 1), consistent with patterns observed in fruits (Sapkota *et al*., 2023). This domestication‐associated gradient is mirrored by combinations of genetic variations at the SlSAMT locus (Figs S3, S4), indicating that natural allelic variation controlling MeSA biosynthesis has been differentially retained during tomato domestication. Rather than reflecting loss of the SA‐MeSA pathway, these patterns suggest that domestication has quantitatively tuned MeSA production by modulating metabolic flux while preserving pathway functionality, highlighting that adaptive changes in plant metabolism often occur through shifts in metabolite abundance rather than through gain or loss of entire pathways (Fernie & Tohge, 2017). During the long‐term co‐evolution between plant roots and soilborne organisms, root‐derived chemical signals are increasingly recognized to exhibit context‐dependent or dual ecological functions (Guerrieri *et al*., 2019). Accordingly, compounds that function in defense in aerial tissues can become liabilities in belowground environments. Consistent with this interpretation, the observed reduction in root MeSA levels during domestication likely reflects shifts in selective pressures associated with the transition from natural ecosystems to managed agroecosystems (Meyer & Purugganan, 2013). In agricultural settings, reduced emission of MeSA may confer an advantage by limiting parasite attraction, suggesting that domestication may have favored genotypes with lower root MeSA levels and enhanced resistance. In this context, the ecological role of root‐emitted volatiles may have shifted during domestication, leading to reduced emission of MeSA, a compound that can be exploited by parasites, while maintaining essential SA‐dependent defense functions.

### Implications for crop improvement and future directions

RKN represents a major constraint on tomato production world‐wide, and the extensive use of chemical nematicides has led to increased resistance of RKN and exacerbated environmental consequences (Grabau *et al*., 2021; Shilpa *et al*., 2022; Poudel *et al*., 2025). From an applied perspective, SlSAMT represents a metabolic susceptibility factor that can be targeted to improve resistance to RKN. Unlike classical resistance (*R*) genes, which often impose strong selection pressure on pathogens (Seifi *et al*., 2011; El‐Sappah *et al*., 2019; Devran *et al*., 2023), modulation of metabolic traits such as MeSA biosynthesis may provide a more durable strategy by reducing host attractiveness rather than directly inhibiting pathogen growth. Our GWAS results indicate that root MeSA accumulation is a polygenic trait, with *SlSAMT* acting as a major, but not exclusive, control point (Tables S2, S3). Additional loci likely contribute to MeSA regulation through modulation of SA metabolism or downstream pathways (Frick *et al*., 2023; Sapkota *et al*., 2023; Lu *et al*., 2025). As such, a comprehensive understanding of the relative contributions of MeSA‐caused susceptibility and SA/MeSA‐based defenses will be critical. Direct measurements of RKN chemotaxis toward transgenic tomato plants with altered MeSA biosynthesis, together with quantification of SA levels, will provide important foundational knowledge for the genetic improvement of MeSA‐centered strategies for enhancing RKN resistance. Important future research directions also include determining how RKNs perceive and respond to plant‐derived MeSA signal, elucidating how MeSA interacts with other rhizosphere components such as beneficial microorganisms and neighboring plants, and assessing whether similar metabolic trade‐offs between defense and susceptibility occur in other crop species.

## Competing interests

None declared.

## Author contributions

FC conceived the project. FC, WW, MS, TH and EvdK designed the experiments. WW, MS and SBZ performed the experiments. All authors contributed to data analysis. WW wrote the first draft and all authors contributed to manuscript revisions.

## Disclaimer

The New Phytologist Foundation remains neutral with regard to jurisdictional claims in maps and in any institutional affiliations.

## Supporting information


**Fig. S1** Root volatile profiling reveals domestication‐associated variation in volatile composition across the tomato Varitome collection.
**Fig. S2** Genome‐wide linkage disequilibrium decay in the tomato Varitome collection.
**Fig. S3** Regional LD association analysis of the chromosome 9 MeSA9.3 locus.
**Fig. S4** Haplotype structure at the chromosome 9 SAMT locus and its association with root MeSA levels.
**Fig. S5** Expression patterns of tomato SAMT‐like and BAMT/BSMT‐like genes during root‐knot nematode infection.
**Table S1** Primer sequences used for amplification of tomato SABATH family genes.
**Table S2** Genome‐wide association loci associated with root methyl salicylate levels in the tomato Varitome collection.
**Table S3** Genome‐wide association study‐identified quantitative trait loci associated with tomato SABATH family members.
**Table S4** Tomato genes of the SA biosynthetic and signaling pathways analyzed for RKN‐related expression.
Please note: Wiley is not responsible for the content or functionality of any Supporting Information supplied by the authors. Any queries (other than missing material) should be directed to the *New Phytologist* Central Office.

## Data Availability

The sequences for the biochemically characterized SABATH methyltransferase reported in this paper have been deposited in the GenBank database: SlBAMT1 (PZ306473) and SlBAMT2 (PZ306474).
