# Supplementary material for: Domestication‐associated reduction of methyl salicylate in tomato root and its significance for resistance to root‐knot nematode
Source: New Phytol. 2026 Jul 29;252(1):414–26. doi: 10.1111/nph.71478 (PMC13539948; doi:10.1111/nph.71478)
Supplement: Supplementary file 1 [file NPH-252-414-s001.pdf]

New Phytologist

***New Phytologist* Supporting Information**

Article title: Domestication-associated reduction of methyl salicylate in tomato root and its significance for resistance to root-knot nematode

Authors: Weijiao Wang (王威姣)[1], Manoj Sapkota[2], Sobhan Bahrami Zadegan[1], Tarek Hewezi[1], Esther van der Knaap[3], and Feng Chen[1,*]

Article acceptance date: 10 July 2026

The following Supporting Information is available for this article:

Fig. S1 Root volatile profiling reveals domestication-associated variation in volatile composition across the tomato Varitome collection.

Fig. S2 Genome-wide linkage disequilibrium (LD) decay in the tomato Varitome collection.

Fig. S3 Regional LD association analysis of the chromosome 9 MeSA9.3 locus.

Fig. S4 Haplotype structure at the chromosome 9 SAMT locus and its association with root MeSA levels.

Fig. S5 Expression patterns of tomato *SAMT*-like and *BAMT/BSMT*-like genes during root-knot nematode infection.

Table S1 Primer sequences used for amplification of tomato SABATH family genes.

Table S2 Genome-wide association loci associated with root methyl salicylate levels in the tomato Varitome collection.

Table S3 Genome-wide association study (GWAS)-identified quantitative trait loci associated with tomato SABATH family members.

Table S4 Tomato genes of the SA biosynthetic and signaling pathways analyzed for RKN-related expression.

Fig. S1 Root volatile profiling reveals domestication-associated variation in volatile composition across the tomato Varitome collection. (**a**) Representative gas chromatography-mass spectrometry (GC-MS) profiles of root volatiles from tomato cv. M82. IS, internal standard. (**b**) Levels of major root-emitted volatile compounds in tomato accessions grouped by major subpopulation. Data represent means ± standard error (SE) from three independent biological replicates. Statistically significant differences among subpopulations are indicated in the boxplots (adjusted $P < 0.05$).

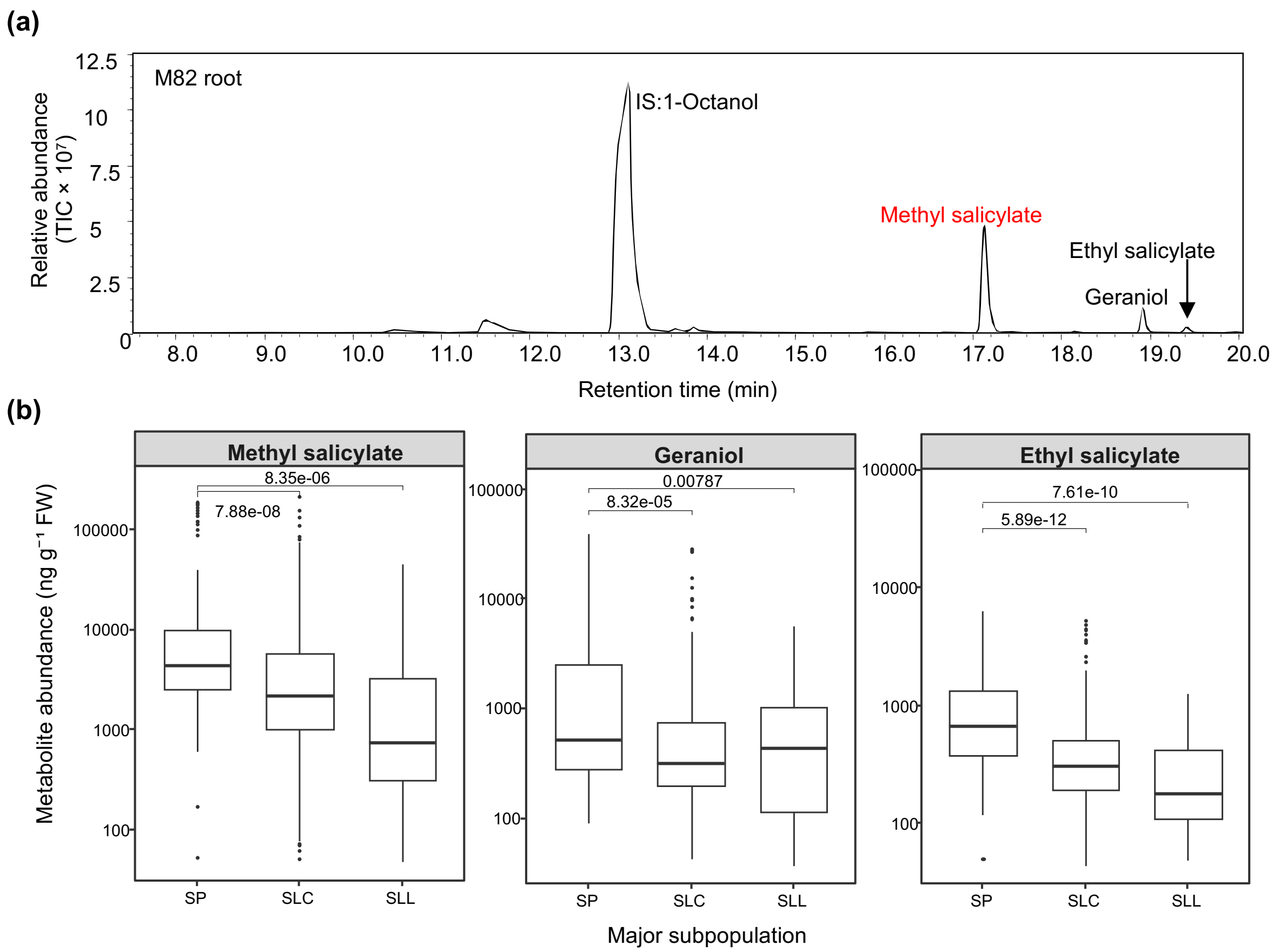

Fig. S2 Genome-wide linkage disequilibrium (LD) decay in the tomato Varitome collection. Genome-wide LD decay based on pairwise SNP comparisons across the Varitome collection. The blue curve shows the decline of linkage disequilibrium (r^2) with increasing physical distance (kb) across the genome. The red dashed line indicates the threshold of $r^2 = 0.10$, corresponding to an estimated average LD decay distance of ~200 kb, defining the effective mapping resolution of GWAS in this population.

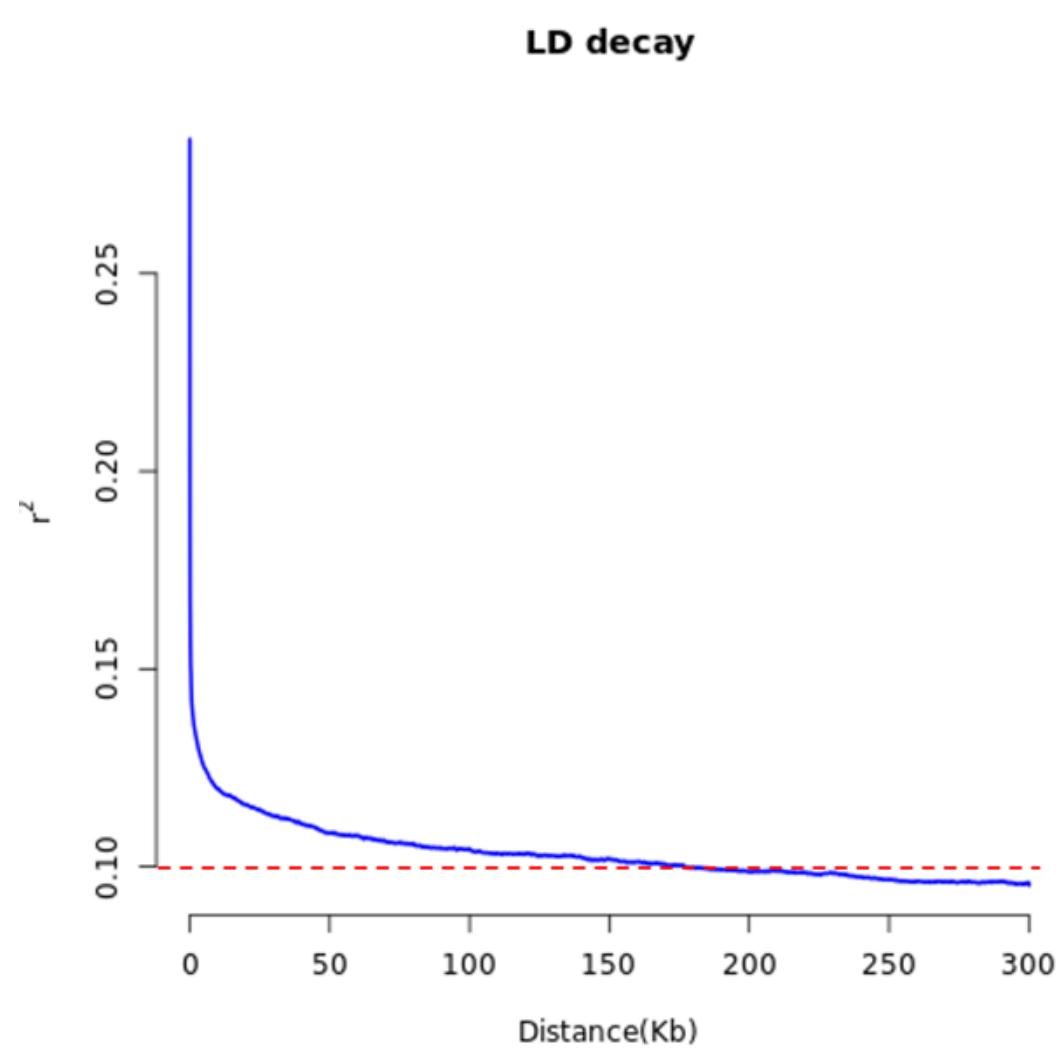

Fig. S3 Regional LD association analysis of the chromosome 9 MeSA9.3 locus. Pairwise LD between lead GWAS variants and neighboring markers was quantified as the squared Pearson correlation coefficient (r^2). Each point represents an individual variant plotted by physical position and r^2 with the lead SV. The red vertical line marks the position of lead variant at the locus and the *SlSAMT* gene position. Two INDELs located ~154 kb upstream of *SlSAMT* showed high LD ($r^2 > 0.5$) with the SV lead variant supporting the potential association between the chromosome 9 QTL and the *SlSAMT* region.

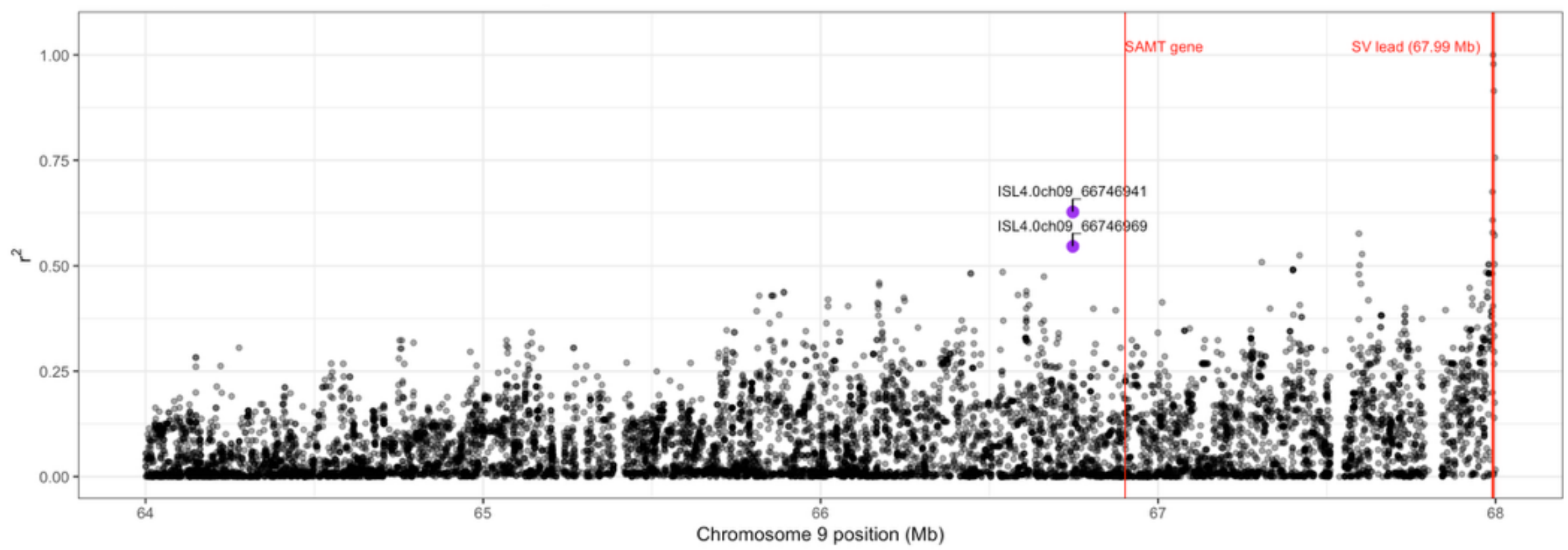

Fig. S4 Haplotype structure at the chromosome 9 *SAMT* locus and its association with root MeSA levels. Left, haplotype heatmap showing allelic variation across *SAMT* locus on chromosome 9 among tomato accessions. Rows represent individual accessions and columns represent genetic variants; colors indicate alternative alleles. Reference allele is represented in blue, alternate in red, heterozygous in yellow and missing data in white. **Right**, distribution of root MeSA levels corresponding to the major haplotypes. 'I' represents wild-enriched haplotype; 'II' represents cultivated-enriched-haplotype. Letters (a, b) denote statistically significant differential MeSA levels (ANOVA, $P = 0.0142$). Distinct haplotypes are associated with high and low MeSA accumulation, consistent with domestication-related allele differentiation.

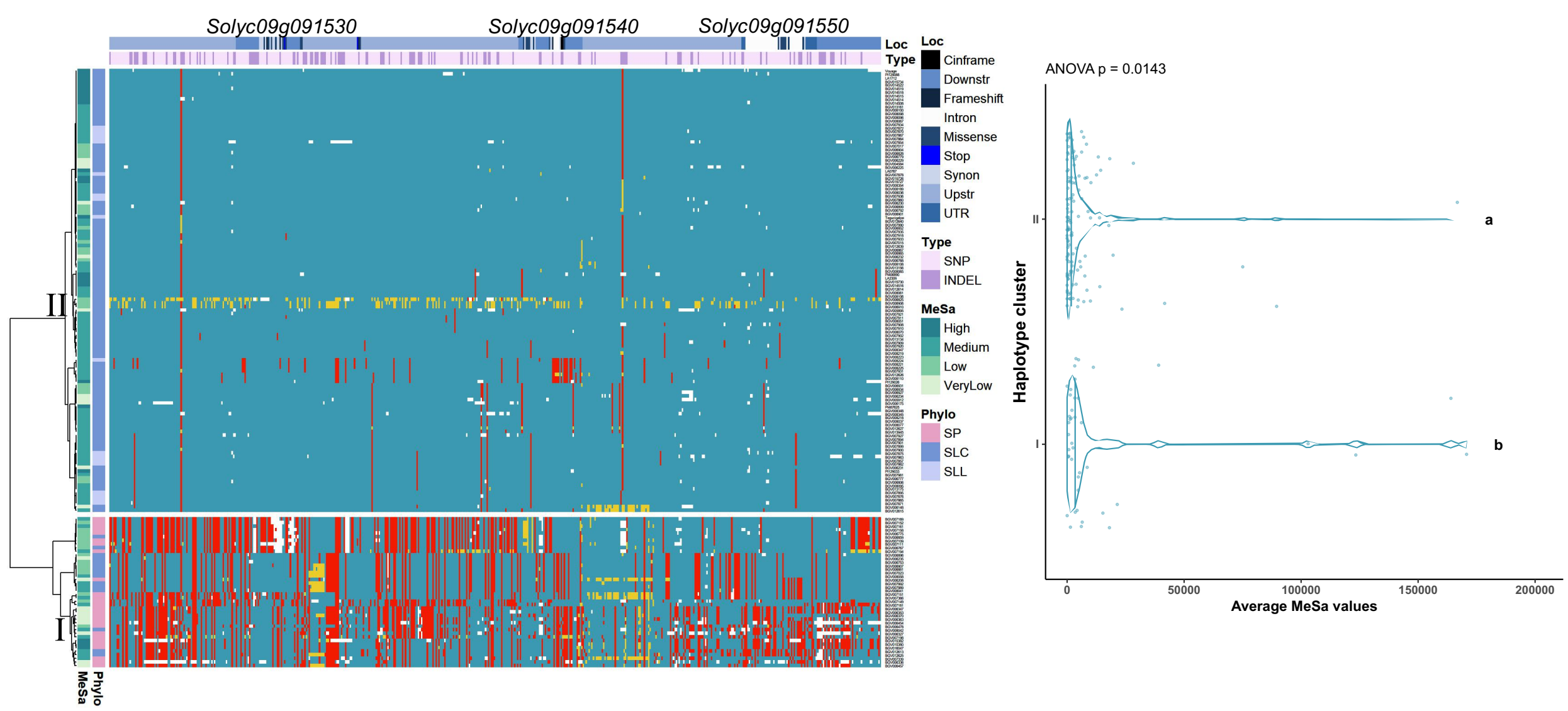

Fig. S5 Expression patterns of tomato *SAMT*-like and *BAMT/BSMT*-like genes during root-knot nematode infection. Top, schematic of the reaction catalyzed by salicylic acid methyltransferase (SAMT), showing methylation of salicylic acid (SA) using S-adenosyl-L-methionine (SAM) as the methyl donor to produce methyl salicylate (MeSA) and S-adenosyl-L-homocysteine (SAH). **Bottom**, heatmap showing $\log_2$(fold change) expression of *SAMT*-like and *BAMT/BSMT*-like genes in galls and neighboring root tissues at 4 and 11 d post-inoculation following *Meloidogyne incognita* infection. Fold changes are calculated relative to uninfected control roots. Asterisks (***) denote statistically significant differential expression (adjusted $P <$ 0.001). Genes highlighted in red indicate significant induction. The color scale represents $\log_2$(fold change) values. Expression values were extracted from a published transcriptome dataset (Ozdemir *et al*., 2024).

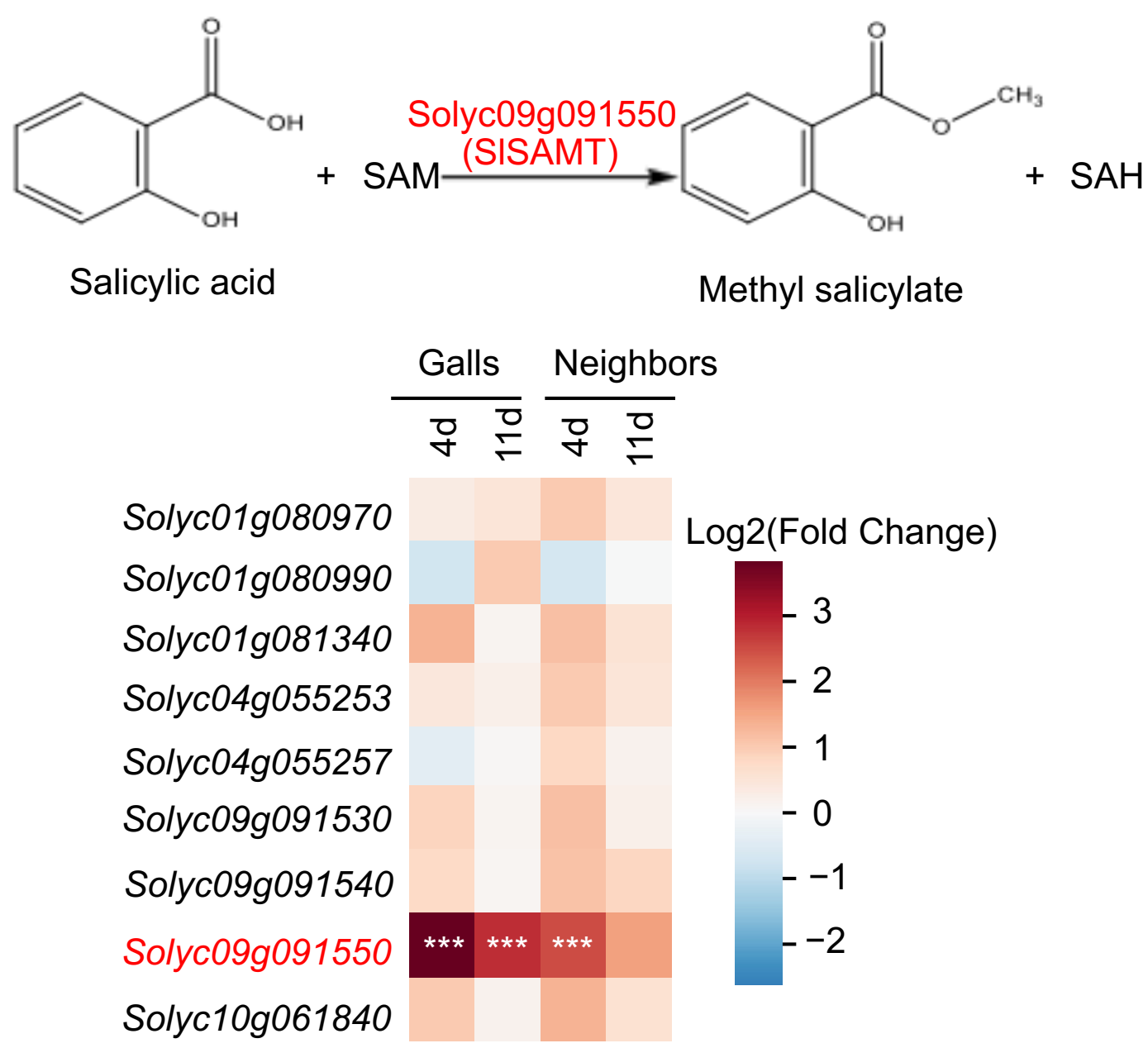

Table S1 ***Primer sequences used for amplification of tomato SABATH family genes.***

Gene ID	Primers
Solyc01g005230.4.1	synthesized
Solyc01g005350.4.1	Forward: GGATCCATGCAGAGAGAAGTGTTAGAG<br>Reverse: CTCGAGTCATTTACGCTTTAAAGCAACA
Solyc01g005360.4.1	synthesized
Solyc01g080970.3.1	Forward: ATGCCTGTTTTAGAAGATGCCATC<br>Reverse: CCGACTGGGAGGTGCTTATT
Solyc01g080990.3.1	Forward: ATGGGAAGGAGCAAAGTTGTG<br>Reverse: TCATTTCTTTCTTAAAGATATGACAATGTTGG
Solyc01g081340.4.1	Forward: ATGGAAGTTGCTAATATTCTTCAC<br>Reverse: TTAAGACTTAATCAAAGAGACTATAACAT
Solyc02g084950.3.1	Forward: ATGGATGTTGAGAAAGTCTTCC<br>Reverse: CTATGGTTTTCGAAGAACGAGAAG
Solyc02g091140.3.1	synthesized
Solyc04g055253.1.1	Forward: ATGGAGGTCACTAAAGTTCTTCAC<br>Reverse: CTAATTTATTTTGGTCAACGAAATAGTGAC
Solyc04g055257.1.1	Forward: ATGGAGGTCACTAGAGTTCTTCAC<br>Reverse: CTAGTTTATTTTGGTCAACGAAACAGTGAC
Solyc09g091530.2.1	Forward: ATGAAGGTTGTAGAAGTTCTTCGT<br>Reverse: TTAGTTGTTTTCTTTGGTCAAAGATACA
Solyc09g091540.2.1	Forward: ATGAAGCTTGTTGAAGTTCTTCAC<br>Reverse: TTAGTTTTTTCTTGTCATGGAGACAG
Solyc09g091550.3.1	Forward: ATGAAGGTTGTTGAAGTTCTTC<br>Reverse: TTATTTTTTCTTGGTCAAGGAGAC
Solyc10g049500.1.1	synthesized
Solyc10g061840.3.1	Forward: ATGGTACTCGAAATTTTTTTACCAATGAC<br>Reverse: TTATTTTTTCCGCAAGCATAAACAAATATTG
Solyc12g014500.3.1	Forward: ATGGCTACATTAGGTGATAAC<br>Reverse: CTACACAAGAGAAAGGGAAGCAAC

Table S2 Genome-wide association loci associated with root methyl salicylate levels in the tomato Varitome collection.

QTLs	SNP	Chr	Pos	LOD	PVE(%)	Type
MeSA1.1	ISL4.0ch01_323684	Ch01	323684	23.32	11.2	INDEL
MeSA1.1	ISL4.0ch01_527390	Ch01	527390	24.56	15.37	INDEL
MeSA1.1	SSL4.0CH01_673024	Ch01	673024	24.23	20.82	SNP
MeSA1.2	ISL4.0ch01_55676207	Ch01	55676207	30.13	21.73	INDEL
MeSA1.3	VSL4.0CH01_71135818	Ch01	71135818	11.96	20.69	SV
MeSA1.4	SSL4.0CH01_75169654	Ch01	75169654	17.41	25.32	SNP
MeSA1.5	VSL4.0CH01_82011370	Ch01	82011370	10.02	8.88	SV
MeSA1.6	VSL4.0CH01_87959944	Ch01	87959944	6.42	6.37	SV
MeSA2.1	SSL4.0CH02_14856084	Ch02	14856084	8.74	3.74	SNP
MeSA3.1	ISL4.0ch03_63655054	Ch03	63655054	14.31	3.57	INDEL
MeSA5.1	SSL4.0CH05_417542	Ch05	417542	9.36	10.32	SNP
MeSA5.2	SSL4.0CH05_2292713	Ch05	2292713	11.71	8.07	SNP
MeSA6.1	VSL4.0CH06_27255850	Ch06	27255850	17.77	31.71	SV
MeSA6.2	ISL4.0ch06_43887623	Ch06	43887623	28.46	4.69	INDEL
MeSA8.1	SSL4.0CH08_2411559	Ch07	2411559	12.53	12.52	SNP
MeSA8.2	SSL4.0CH08_56315523	Ch08	56315523	8.15	14.52	SNP
MeSA9.1	VSL4.0CH09_5251180	Ch09	5251180	6.71	1.45	SV
MeSA9.2	ISL4.0ch09_64449339	Ch09	64449339	20.9	11.95	INDEL
MeSA9.3	VSL4.0CH09_67992261	Ch09	67992261	6.92	2.94	SV
MeSA10.1	ISL4.0ch10_20399990	Ch10	20399990	20.42	14.98	INDEL
MeSA10.2	VSL4.0CH10_45092277	Ch10	45092277	6.69	8.05	SV
MeSA10.3	ISL4.0ch10_54551725	Ch10	54551725	8.24	0.01	INDEL
MeSA10.4	VSL4.0CH10_56257618	Ch10	56257618	6.37	5.65	SV
MeSA10.4	ISL4.0ch10_56367599	Ch10	56367599	24.36	9.42	INDEL
MeSA10.5	ISL4.0ch10_58411737	Ch10	58411737	15.75	6.53	INDEL
MeSA11.1	ISL4.0ch11_2441594	Ch11	2441594	8.72	0.27	INDEL
MeSA12.1	VSL4.0CH12_3887210	Ch12	3887210	10.09	9.77	SV

Footnotes: MeSA, methyl salicylate; QTL, quantitative trait locus; SNP, single-nucleotide polymorphism; Chr, chromosome; Pos, physical position (bp) on the chromosome; LOD, logarithm of odds; PVE, percentage of phenotypic variance explained; Type, variant type; SV: structural variant; INDEL: insertions and deletions.

Table S3 ***Genome-wide association study (GWAS)-identified quantitative trait loci associated with tomato SABATH family members.***

Gene ID	Position	Nearby GWAS QTL
**Solyc01g005230.4.1**	SL4.0ch01:195,535..197,081	MeSA1.1
**Solyc01g005350.4.1**	SL4.0ch01:262,224..269,870	MeSA1.1
**Solyc01g005360.4.1**	SL4.0ch01:272,007..273,422	MeSA1.1
**Solyc01g080970.3.1**	SL4.0ch01:72,878,814..72,880,243	MeSA1.3
**Solyc01g080990.3.1**	SL4.0ch01:72,887,921..72,889,634	MeSA1.3
**Solyc01g081340.4.1**	SL4.0ch01:73,164,662..73,166,396	MeSA1.3
**Solyc02g084950.3.1**	SL4.0ch02:46,000,606..46,003,189	
**Solyc02g091140.3.1**	SL4.0ch02:50,587,186..50,588,998	
**Solyc04g055253.1.1**	SL4.0ch04:53,193,819..53,208,562;	
**Solyc04g055257.1.1**	SL4.0ch04:53,212,157..53,228,812	
**Solyc04g080660.4.1**	SL4.0ch04:62,741,768..62,746,054	
**Solyc07g064990.3.1**	SL4.0ch07:66,759,476..66,762,001	
**Solyc09g091530.2.1**	SL4.0ch09:66,886,423..66,888,884	MeSA9.3
**Solyc09g091540.2.1**	SL4.0ch09:66,892,779..66,894,873	MeSA9.3
**Solyc09g091550.3.1**	SL4.0ch09:66,901,227..66,903,818	MeSA9.3
**Solyc10g049500.1.1**	SL4.0ch10:45,063,396..45,065,950	MeSA10.1
**Solyc10g061840.3.1**	SL4.0ch10:21,470,674..21,472,218	
**Solyc12g014500.3.1**	SL4.0ch12:5,502,198..5,509,673	

Footnotes: MeSA, methyl salicylate; QTL, quantitative trait locus.

New Phytologist

Table S4 Tomato genes of the SA biosynthetic and signaling pathways analyzed for RKN-related expression.

Name	Gene ID	References
SlPAL5	*Solyc09g007920*	(Guo & Wang, 2009; Zhang *et al*., 2023)
*SlBEBT-like1**	*Solyc07g049655*	(Wang, Y *et al*., 2025)
*SlBEBT-like2**	*Solyc07g049645*	(Wang, Y *et al*., 2025)
SlBBH	*Solyc03g122350*	(Wang, Y *et al*., 2025)
SlBSE	*Solyc02g069800*	(Wang, Y *et al*., 2025)
SlSAMT	*Solyc09g091550*	Current study and (Tieman *et al*., 2010)
SlMES1	*Solyc02g065240*	(Frick *et al*., 2023; Lu *et al*., 2025)
SlNPR1	*Solyc07g040690*	(Li *et al*., 2019)
SlTGA1	*Solyc04g011670*	(Zheng *et al*., 2025)
SlPR1	*Solyc01g106620*	(Chen *et al*., 2023)
SlWRKY16	*Solyc07g056280*	(Kumar *et al*., 2023)
SlWRKY31	*Solyc05g053380*	(Kumar *et al*., 2023)
SlWRKY45	*Solyc08g067360*	(Chinnapandi *et al*., 2017; Huang *et al*., 2022)
SlWRKY80	*Solyc03g095770*	(Nie *et al*., 2023; Chen *et al*., 2024)

Footnotes: Gene identifiers are based on the SL4.0/ITAG4.0 tomato genome annotation. * The SlBEBT gene ID reported by Wang, Y *et al*. (2025), *Solyc07g049660*, is not present in SL4.0/ITAG4.0. Sequence matching identified two 100% identical SL4.0 candidates, *Solyc07g049655* and *Solyc07g049645*, designated here as *SlBEBT-like1* and *SlBEBT-like2*, respectively. Expression values shown in Fig. 7 were extracted from a published transcriptome dataset (Ozdemir *et al*., 2024). Abbreviation: SA, salicylic acid; RKN, root-knot nematode; Sl, *Solanum lycopersicum*; PAL5, phenylalanine ammonia-lyase 5; BEBT, benzoyl-CoA:benzyl alcohol benzoyltransferase; BBH, benzylbenzoate hydroxylase; BSE, benzylsalicylate esterase; SAMT, salicylic acid methyltransferase; MES1, salicylic acid methyl esterase 1; NPR1, nonexpressor of pathogenesis-related gene 1; TGA1, TGA[C/T]G motif-binding transcription factor 1; PR1, pathogenesis-related protein 1; WRKYs, WRKY transcription factors.

References

Chen N, Shao Q, Xiong Z. 2023. Isolation and characterization of a pathogenesis-related protein 1 (*SlPR1*) gene with induced expression in tomato (*Solanum lycopersicum*) during *Ralstonia solanacearum* infection. *Gene* **855**: 147105.

Chen Y, Wang Z, Nie W, Zhao T, Dang Y, Feng C, Liu L, Wang C, Du C. 2024. Study on the function of *SlWRKY80* in tomato defense against *Meloidogyne incognita*. *Int. J. Mol. Sci.* **25**(16).

Chinnapandi B, Bucki P, Braun Miyara S. 2017. *SlWRKY45*, nematode-responsive tomato *WRKY* gene, enhances susceptibility to the root knot nematode; *M. javanica* infection. *Plant Signal. Behav.* **12**(12): e1356530.

Frick EM, Sapkota M, Pereira L, Wang Y, Hermanns A, Giovannoni JJ, Van Der Knaap E, Tieman DM, Klee HJ. 2023. A family of methyl esterases converts methyl salicylate to salicylic acid in ripening tomato fruit. *Plant Physiol.* **191**(1): 110-124.

Guo J, Wang M-H. 2009. Characterization of the phenylalanine ammonia-lyase gene (*SlPAL5*) from tomato (*Solanum lycopersicum* L.). *Mol. Biol. Rep.* **36**(6): 1579-1585.

Huang H, Zhao W, Qiao H, Li C, Sun L, Yang R, Ma X, Ma J, Song S, Wang S. 2022. SlWRKY45 interacts with jasmonate-ZIM domain proteins to negatively regulate defense against the root-knot nematode *Meloidogyne incognita* in tomato. *Hortic. Res.* **9**.

Kumar A, Sichov N, Bucki P, Miyara SB. 2023. SlWRKY16 and SlWRKY31 of tomato, negative regulators of plant defense, involved in susceptibility activation following root-knot nematode *Meloidogyne javanica* infection. *Sci. Rep.* **13**(1): 14592.

Li R, Liu C, Zhao R, Wang L, Chen L, Yu W, Zhang S, Sheng J, Shen L. 2019. CRISPR/Cas9-Mediated *SlNPR1* mutagenesis reduces tomato plant drought tolerance. *BMC Plant Biol.* **19**(1): 38.

Lu Y, Li J, Cheng K, Zhu G, Zhu B, Fu D, Qu G, Luo Y, Ma L, Lin T, et al. 2025. SlMES1 modulates methyl salicylate to influence fruit volatile profiles in tomato. *Plant Physiol. Biochem.* **221**: 109561.

Nie W, Liu L, Chen Y, Luo M, Feng C, Wang C, Yang Z, Du C. 2023. Identification of the regulatory role of *SlWRKYs* in tomato defense against *Meloidogyne incognita*. *Plants (Basel)* **12**(13).

Ozdemir S, Piya S, Lopes-Caitar VS, Coffey N, Rice JH, Hewezi T. 2024. Local and systemic transcriptome and spliceome reprogramming induced by the root-knot nematode *Meloidogyne incognita* in tomato. *Hortic. Res.*: uhae206.

Tieman D, Zeigler M, Schmelz E, Taylor MG, Rushing S, Jones JB, Klee HJ. 2010. Functional analysis of a tomato salicylic acid methyl transferase and its role in synthesis of the flavor volatile methyl salicylate. *Plant J.* **62**(1): 113-123.

Wang Y, Song S, Zhang W, Deng Q, Feng Y, Tao M, Kang M, Zhang Q, Yang L, Wang X, et al. 2025. Deciphering phenylalanine-derived salicylic acid biosynthesis in plants. *Nature* **645**(8079): 208-217.

Zhang F, Wang J, Li X, Zhang J, Liu Y, Chen Y, Yu Q, Li N. 2023. Genome-wide identification and expression analyses of phenylalanine ammonia-lyase gene family members from tomato *(Solanum lycopersicum*) reveal their role in root-knot nematode infection. *Front. Plant Sci.* **14**: 1204990.

Zheng Q, Meng X, Fan X, Chen S, Sang K, Yu J, Zhou Y, Xia X. 2025. Regulation of *PILS* genes by bZIP transcription factor TGA7 in tomato plant growth. *Plant Sci.* **352**: 112359.
